# Study about photoinduction-based technology for trapping small brown planthopper

**DOI:** 10.3389/fpls.2022.905001

**Published:** 2022-08-29

**Authors:** Shaoqi Zhan, Zhentao Sheng, Yiming Liu, Ke Xu, Jiahui Wang, Weixing Cao, Yongchao Tian, Jun Ni

**Affiliations:** ^1^College of Agriculture, Nanjing Agricultural University, Nanjing, China; ^2^National Engineering and Technology Center for Information Agriculture, Nanjing, China; ^3^Engineering Research Center of Smart Agriculture, Ministry of Education, Nanjing, China; ^4^Collaborative Innovation Center for Modern Crop Production co-sponsored by Province and Ministry, Nanjing, China

**Keywords:** rice planthopper, light trapping, light spectrum, light intensity, shape, phototaxis

## Abstract

To provide a theory to guide the selection of the illumination parameters of light emitting diode (LED)-based light sources used for trapping *Laodelphax striatellus*, we used LED light sources and devices built in-house to detect *L. striatellus* phototactic behavior. Through phototaxis screening experiments of different light sources and the comparative experimental method, we analyzed the response patterns of *L. striatellus* to wavelength, light intensity, layout, flash frequency of monochromatic light sources, as well as combined color light sources, and discussed the mechanisms of the phototactic behavior of *L. striatellus* under different light sources. The results of the monochromatic light experiment showed that the trapping rate of the *L. striatellus* to the linear blue light source of 460 nm was the highest and was also significantly affected by the light intensity. The results of the experiments with the combined color light sources showed that compared with the linear 460 nm blue light source, the trapping rate of the *L. striatellus* was significantly improved by the polychromatic light, and the blue–green light led to the best improvement, reaching 1.5 times that of the trapping rate in the case of monochromatic light sources. The wavelength composition, light intensity, shape, and flash frequency of the light source used in this study can provide a theoretical basis for the development of LED-based light traps specifically for *L. striatellus*.

## Introduction

As one of the three major pests in rice fields, the *Laodelphax striatellus* not only damage to phloem to suck phloem sap but also transmits diseases such as rice stripe blight, rice black-streaked dwarf disease, and southern black-streaked dwarf disease, causing serious damage to rice yield and quality (Yang et al., 2014). According to incomplete statistics, during the periods of high incidence of pests in the rice area in Asia, areas damaged by *L. striatellus* exceeded 20 million hectares, and the losses reached one million tons even after prevention and control, making the *L. striatellus* the most serious pest threat to food production security in the world (Heong et al., 2015; Hong et al., 2017). At present, *L. striatellus* control mainly relies on chemical pesticides (Cao et al., 2020), but the 3R (resistance, resurgence, residue) problem caused by the long-term use of pesticides is becoming increasingly serious, leading to food safety risk, environmental pollution, and other problems that seriously endanger people’s daily life. Therefore, there is an urgent need to develop precise, efficient, green, and pollution-free control of rice field pests for rice production.

Pest light trapping technology is a method that uses insect phototactic behavior to lure insects to gather at a fixed location for centralized eradication. This is a powerful and green pest control technology that is safe, environmentally friendly, efficient, free of pesticide residue, and does not lead to resistance (Sang et al., 2019). However, in the existing control process, the design and application of the light sources for trapping are oversimplified, without considering the response of the insect visual system to different light stimuli, thus resulting in a poor trapping performance. The response of insects to light is strongly affected by the intensity of radiation, the shape and contrast of the radiation source and the physiological state of the insect (Ben-Yakir et al., 2013). It was found that insects have significantly different selectivity for light sources of different characteristics (Park and Lee, 2017). The wavelength, light intensity, shape, and other factors of light sources significantly affect the phototropic behavior of insects. Kim found that the green (520 nm) LED light resulted in the highest phototactic response from the M. separata adults (Kim et al., 2018). There are three main trends in insect phototropism as light intensity changes: “S” curve, inverse relationship and positive relationship (Chen et al., 2012). Different shapes of sticky board traps based on the same material composition showed significant differences in the number of trapped whiteflies, with cylindrical sticky boards capturing significantly more whiteflies than other shapes of sticky boards (Byrne et al., 1986). Green LEDs and green-violet combined LED light sources had significantly higher trapping rate for whiteflies compared to stick insect swatches, while other wavelengths or combined wavelengths had significantly lower trapping rate than stick insect swatches (Stukenberg et al., 2015).

Behavioral and physiological studies on the light trapping of *L. striatellus* have shown that under monochromatic light, the trapping rate of *L. striatellus* to blue light sources is the highest (Yang et al., 2014). However, there are few reports on whether there are differences in the phototactic behavior of *L. striatellus* to different blue lights and different combinations of lights, and whether the light intensity, shape of the light source, and flash frequency of the light source affect the phototactic behavior of *L. striatellus* (Kim et al., 2019). In this study, based on the characteristics of the *L. striatellus*’s visual system and activity pattern, we optimized the illumination parameters of light sources for trapping *L. striatellus* and explored the factors that affect the *L. striatellus*’s phototaxis. Our study is of great significance in the design and development of *L. striatellus* light traps.

Due to their narrow wavelength band and low power consumption, LEDs are often the best choice for light sources used in insect traps (Cohnstaedt et al., 2008; Gi and Seon, 2012; Kim et al., 2019). In our study, we used LED lights and a device built-in house that measures *L. striatellus* phototactic behaviors to perform phototaxis screening experiment with different light sources. The study is summarized as follows. 1. With the same light intensity in the blue light band (440 nm–500 nm) to which *L. striatellus* are effective, we set a finer wavelength interval and conducted a monochromatic light screening experiment to identify the most effective monochromatic light wavelength for the *L. striatellus*. 2. Based on the optimized monochromatic sensitive wavelength, we conducted experiments on the phototaxis of *L. striatellus* under different light intensities, shapes, and flash frequencies and further analyzed the differences in the trapping rates of the *L. striatellus* under the different parameters of the light sources. 3. Based on the optimized parameters of the light sources, we tested the phototaxic response of *L. striatellus* to different polychromatic lights to analyze the effects of the different polychromatic illumination on the phototactic behavior of the *L. striatellus*.

## Materials and methods

### Insect material

**FIGURE 1:**
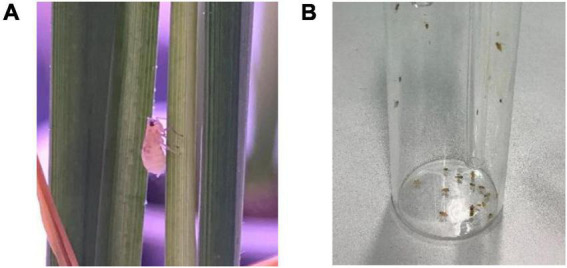
**(A)** Individual of Laodelphax striatellus on rice plant, **(B)** small planthopper sucker.

The experiments used healthy adult insects of laboratory-reared *Laodelphax striatellus*, with a body length of 2.3 mm to 4.0 mm, as shown in Figure 1. It is one of the three major planthoppers species (*Laodelphax striatellus*, *Sogatella furcifera*, and *Nilaparvata lugens*) that damage rice in China. They are widespread and a representative pest species. Before the experiments, the planthoppers were placed in a transparent rearing box. The temperature in the transparent rearing box is controlled at 24–27°C, humidity is controlled at 70%, photoperiod 16L:8D. In each experiment, a small planthopper sucker (Figure 1) was used to suck them in and to count them. Through the transparent glass container, it was observed that individual planthoppers were very active after being sucked into the container, indicating suitability for the phototaxis experiments.

### Insect behavioral response box

**FIGURE 2:**
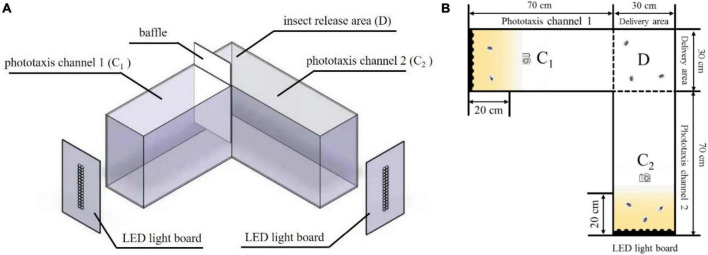
Insect behavioral response box. C_1_, phototaxis channel 1; C_2_, phototaxis channel 2; D, insect release area. **(A)** Three-dimensional structure diagram, **(B)** Plane structure drawing.

The insect behavioral response box used for the *L. striatellus* is shown in Figure 2. The device was divided into three areas: insect release area D (30 cm × 30 cm × 50 cm), phototaxis channel C_1_ (30 cm × 70 cm × 50 cm), and phototaxis channel C_2_ (30 cm × 70 cm × 50 cm). LED light sources were placed at the end of the two phototaxis channels. The reaction box was designed to be L-shaped to avoid mutual influence of the light sources in the two channels during the phototaxis experiments. A baffle was set up at the intersection of the C_1_ and D areas. When the baffle was pushed down, only the LED light source in C_2_ was turned on to conduct single-channel experiments. When the baffle was pulled up, the LED light sources in the two channels were turned on at the same time to allow for dual-channel experiments. The areas within 20 cm of the LED light source (the yellow areas in the middle right of Figure 2) were used as the phototaxis areas. The number of insects in a phototaxis area divided by the total number of insects was used to represent the trapping rate of *L. striatellus* to that light source, namely, the degree of response. The higher the trapping rate, the greater the response of the *L. striatellus* to the light source, and vice versa. To avoid the effect of light reflection on the experiments, the outer and inner walls of the entire box were made of black frosted acrylic. The top surface was made of transparent acrylic material allowing for observation. An infrared camera was installed on the top surface of the two channels at a distance of 30 cm from the light source to record the test process.

### Experimental light source

**FIGURE 3:**
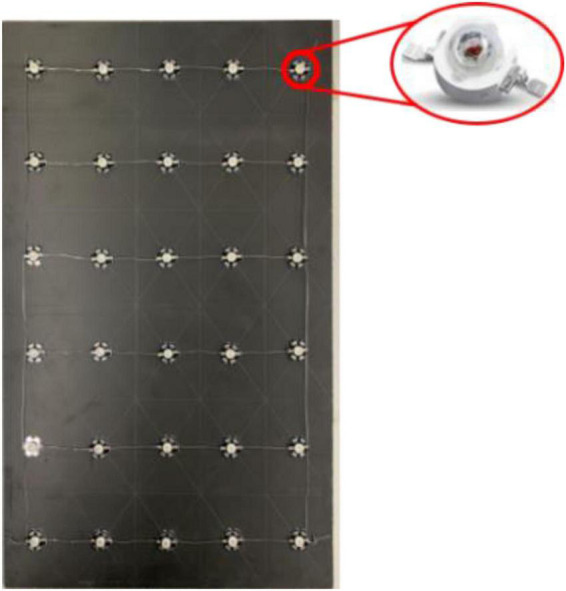
LED light board with gigh-power LEDs.

The light source used in the experiments consisted of a light source array composed of several high-power LEDs (1 W/unit) and a rectangular foam board (30 × 50 cm), as shown in Figure 3. The LED lamp had a diameter of 8 mm, a light-emitting angle of 60°, and a full spectral peak width at half height of ±10 nm. To avoid the heating effect of the LED light source, a small heat sink with a diameter of 20 mm was placed beneath each LED. The LEDs were powered by direct current power supplies. The LED light intensity was changed by adjusting the power supply current. The light intensity at the intersection of areas C_1_, C_2_, and D (70 cm from the light source) was adjusted using a digital spectral illuminance meter (Aicevoos AS-V10).

### Experimental method

The experiments used to study the responses to wavelength, light intensity, shape, flash frequency, and combined light were set up according to the phototaxis characteristics of *L. striatellus*. Following the above sequence, the experiment of each step was conducted on the basis of the experimental results of the previous step. According to the nocturnal living habits of *L. striatellus*, the phototaxis experiments were conducted during their active period (19:00–23:00) in an indoor dark environment at constant temperatures between 24 and 27°C and a humidity of 70%. Before experiment, the LED light source is placed at the end of the convergence channel and the light intensity is calibrated using a digital spectral illuminance meter(Aicevoos AS-V10). Then sucked *L. striatellus* are released in the insect release area (D), the box is closed to ensure that they do not escape, and then the light source is turned on for the phototropism test. To ensure the stability and reliability of the data, four replicate trials were conducted for each experiment, and 50 *L. striatellus* were released in each trial, for a total of 200 insects in each experiment. After an illumination time of 30 min (all tests), we counted the number of insects in the phototaxis areas of the two channels, and calculated the trapping rates.

### Phototaxis of *Laodelphax striatellus* in response to wavelength

**FIGURE 4:**
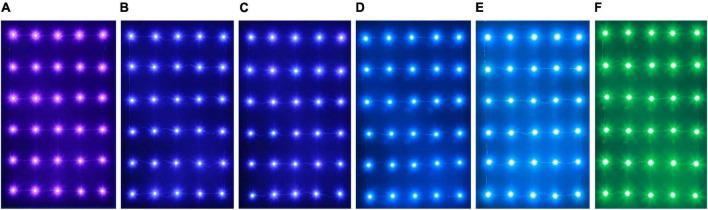
LED light sources with different wavelengths. **(A)** 440 nm, **(B)** 450 nm, **(C)** 460 nm, **(D)** 470 nm, **(E)** 480 nm, **(F)** 500 nm.

The experiment was divided into two parts: the single-channel phototaxis experiment and the dual-channel comparison experiment. The single-channel experiment aimed to elucidate the maximum phototaxis of *L. striatellus* to light sources with different wavelengths. The dual-channel experiment aimed to further compare light sources with similar trapping rates. The first test was the single-channel phototaxis experiment. In the blue light bands that *L. striatellus* are most sensitive to, six spectral bands, 440, 450, 460, 470, 480, and 500 nm, were used to conduct the wavelength response experiment of the phototaxis of *L. striatellus*. The light source consisted of a 5*6 LED array, as shown in Figure 4. Before the experiment started, the light intensity of the light source at different wavelengths was adjusted to 500 lux (70 cm from the light source). After the single-channel phototaxis experiment, the trapping rates at different wavelengths were calculated statistically. The dual-channel experiment for light sources with similar trapping rates was then conducted. The results of the single-channel and dual-channel experiments were analyzed to determine the wavelength characteristics of the phototaxis response of *L. striatellus*.

### Phototaxis of *Laodelphax striatellus* in response to light intensity

**FIGURE 5:**
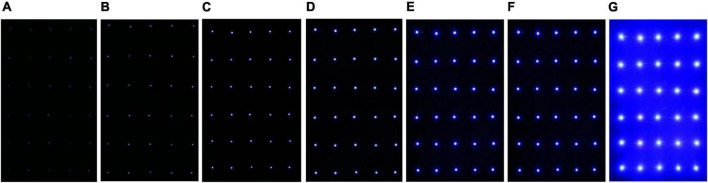
LED-light sources with different light intensities. **(A)** 0.001 lux, **(B)** 0.01 lux, **(C)** 0.1 lux, **(D)** 10 lux, **(E)** 100 lux, **(F)** 1000 lux, **(G)** 2000 lux.

Based on the results of “Phototaxis of L. striatellus in response to wavelength”, we conducted the light intensity response experiment for the phototropism behavior of *L. striatellus*. The light intensity response experiment was a single-channel experiment. Seven light intensities of 0.001, 0.01, 0.1, 10, 100, 1,000, and 2,000 lux were used to conduct the light intensity response experiment. The light source at each light intensity was composed of a 5*6 LED array, as shown in Figure 5. At the beginning of the experiment, the baffle between the two phototaxis channels was closed, the light source was placed at the end of C_2_, and the light intensity required for the experiment was obtained by adjusting the current according to the light intensity measurement results of the digital spectral illuminance meter (Aicevoos AS-V10). Then, *L. striatellus* were released from the insect release area and the light source was turned on for light stimulation. By analyzing the variation in the trapping rate with light intensity, we determined the light intensity response characteristics of *L. striatellus* phototactic behavior.

### Phototaxis of *Laodelphax striatellus* in response to light source shape

**FIGURE 6:**
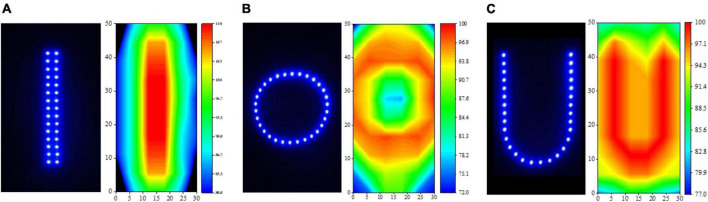
Three shapes of light source and their light intensity distributions. **(A)** linear, **(B)** circular, **(C)** U-shaped.

Based on the results of “Phototaxis of L. striatellus in response to wavelength”, “Phototaxis of L. striatellus in response to light intensity”, we conducted the light-shape response experiment for the phototropism behavior of *L. striatellus*. The experiment was divided into two parts: the single-channel phototaxis experiment and the dual-channel comparison experiment. The single-channel experiment aimed to elucidate the maximum phototaxis of *L. striatellus* to light sources with different shape. The dual-channel experiment aimed to further compare light sources with similar trapping rates. The first test was the single-channel phototaxis experiment, choosing the wavelength that led to the highest trapping rate obtained in section “Phototaxis of L. striatellus in response to light intensity”. Three common light source shapes—U-type, Round, Line-Type—were used to test the phototactic behavior response of *L. striatellus* to the light source shapes. A light source in each shape had a 30 LEDs, as shown in Figure 6, to ensure that the actual shape of the light field distribution in the convergence channel is consistent with the shape of the designed light source. The light intensity distribution of the light field formed by different light source shapes was measured at a distance of 70 cm from the LED light source in the phototaxis channel using a digital spectrophotometer. The measurement method is as follows: divide the luminous section (30 cm × 50 cm) into 5 cm × 5 cm grids at a distance of 70 cm from the light source, measure the light intensity in each grid, and draw the light intensity distribution diagram, as shown in Figure 6. The results showed that the light intensity distribution of the light field was consistent with the shape of the LED and thus suitable for the experiment. At the same voltage and current, the light sources of different shapes operated at the same power, thereby ensuring that the total light intensities in the phototaxis channel were consistent. The light source was then turned on for phototropism experiments, and the results of the single-channel and dual-channel comparison tests were analyzed comprehensively to clarify the phototaxis response of *L. striatellus* to different light source shapes.

### Phototaxis of *Laodelphax striatellus* in response to flash frequency

Based on the results of “Phototaxis of L. striatellus in response to wavelength”, “Phototaxis of L. striatellus in response to light intensity”, “Phototaxis of L. striatellus in response to light source shape” and “Phototaxis of L. striatellus in response to light source shape”, we conducted the light source flash frequency response experiment for the phototropism behavior of *L. striatellus*, and the experiment was a single-channel test. Based on the approach of simulating the strobe light-emitting mode of fireflies, we used six flash frequencies of 1/1200, 1/360, 1/60, 5, 10, and 20 Hz to determine the flash frequency response characteristics of the phototaxis of *L. striatellus* (Zhou, 2009). The lower flash frequency group consisted of 1/1200, 1/360, and 1/60 Hz. The higher flash frequency group consisted of 5, 10, and 20 Hz. The light sources with different flash frequencies all had 30 LEDs with a light intensity of 100 lux. The flash control was implemented by an Arduino UNO microcontroller.

### Phototaxis of *Laodelphax striatellus* in response to combined light

**FIGURE 7:**
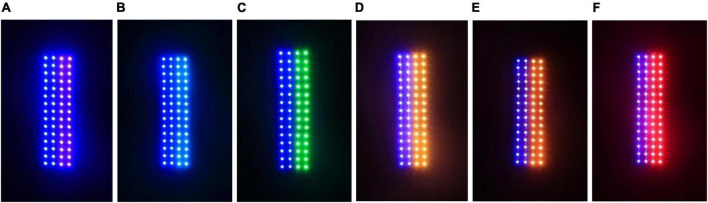
LED light sources with different wavelengths. **(A)** blue–violet, **(B)** blue–cyan, **(C)** blue–green, **(D)** blue–yellow, **(E)** blue–orange, **(F)** blue–red.

Based on the response characteristics of the *L. striatellus* to monochromatic light, we combined different colors of visible light to conduct a combined light response test of the phototaxis behavior of *L. striatellus*. The optimization experiment for the polychromatic light combination was divided into a single-channel experiment and a dual-channel experiment. The single-channel experiment aimed to elucidate the maximum phototaxis of *L. striatellus* to the polychromatic light. The dual-channel experiment aimed to further compare light sources with similar trapping rates. The first test was the single-channel phototaxis experiment. We used six combined light sources of blue–violet, blue–cyan, blue–green, blue–yellow, blue–orange, and blue–red and used monochromatic blue light as a control light source. Each light source with two color combinations consisted of 30 blue LEDs and 30 LEDs of the other color, as shown in Figure 7. By adjusting the current, the light of each color had an intensity of 100 lux. The intensity of the blue control light was set to 200 lux to ensure that the total light intensity was consistent during the experiment. The results of the single-channel and dual-channel experiments were analyzed to determine the phototaxis response of *L. striatellus* to polychromatic light characteristics.

### Data processing

The experimental data were statistically analyzed using Excel and Origin 2021 data processing software. The significance of the differences between different light sources was evaluated by F tests. The least significant difference (LSD) was used in multiple analysis. The trapping rate T_1_ of the *L. striatellus* was used to reflect the phototaxis response of the *L. striatellus* to a light source. The phototaxis ratio T_2_ was used to reflect the difference in the response of the *L. striatellus* to different light sources in the two channels. Both are expressed as the mean ± standard error. The equations are:


(1) $$T\,1=\frac{m_{1}}{n}\times 100\%$$

(2) $$T\,2=\frac{m_{1}}{m_{2}}\times 100\%$$

where m_1_ is the number of *L. striatellus* in the area within 20 cm of the light source in C_1_, m_2_ is the number of the *L. striatellus* in the area within 20 cm of the light source in C_2_, and n is the total number of insects.

## Results and analysis

### Analysis of wavelength response patterns of *Laodelphax striatellus* phototactic behavior

**FIGURE 8:**
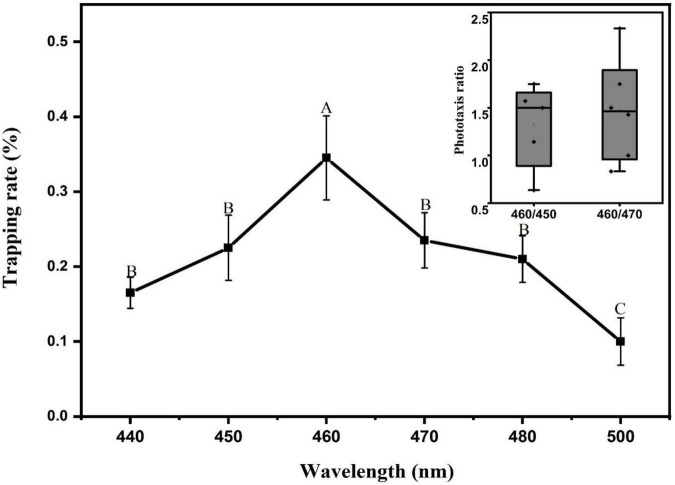
Trapping rate of *L. striatellus* at different monochromatic light in the range 440-500 nm. Each value represents the trapping rate T_1_ of adult *L. striatellus* for the specific wavelength of the light source, and the error bars represent the standard error. Different letters above each group of data indicate significant differences between treatments (*p* < 0.01). The upper right panel shows the trapping rate T2 of 460 nm vs. 470 nm and 460 nm vs. 450 nm.

The experimental results are shown in Figure 8. Under the stimulation of different blue light sources ranging from 440 nm to 500 nm, the *L. striatellus* exhibited phototactic behavior responses. The F test showed that the trapping rates differed significantly between different blue light spectra (df = 5,18; *F* = 4.526; *P* = 0.007). The *L. striatellus* had the best phototaxis response to 460 nm, with a trapping rate of 34.5%, which was significantly higher than that for the other wavelengths. To further compare the responses of the *L. striatellus* to different blue lights, three wavelength bands with the most similar trapping rates, 450 nm, 460 nm, and 470 nm, were selected for the comparison experiments. The trapping rate of *L. striatellus* at 460 nm was 1.3 times that at 450 nm and 1.5 times that at 470 nm.

### Analysis of the light intensity response pattern of *Laodelphax striatellus* phototactic behavior

**FIGURE 9:**
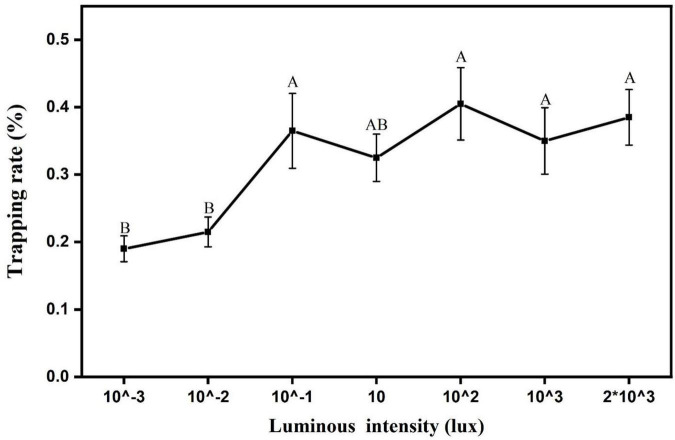
Trapping rate of *L. striatellus*s at different light intensities. Each value represents the trapping rate T_1_ of the adult *L. striatellus* for that light intensity, and the error bars represent the standard error. Different letters above each group of data indicate significant differences between treatments (*p* < 0.01).

The phototaxis light intensity threshold of the *L. striatellus* was measured based on the optimal light source wavelength (460 nm). The experimental results are shown in Figure 9. In this experiment, light sources with seven light intensities were used, all of which could induce phototaxis response behavior of *L. striatellus*. The results of the F test showed that the trapping rates of *L. striatellus* differed significantly between different light intensities (df = 6,21; *F* = 27; *P* = 0.007). The trapping rate of the *L. striatellus* increased with increasing light intensity at low light intensities. When the light intensity exceeded 0.1 lux, an increase in the light intensity of the light source no longer had a significant effect on the phototaxis of the *L. striatellus*.

### Analysis of the shape response pattern of the phototactic behavior of *Laodelphax striatellus*

**FIGURE 10:**
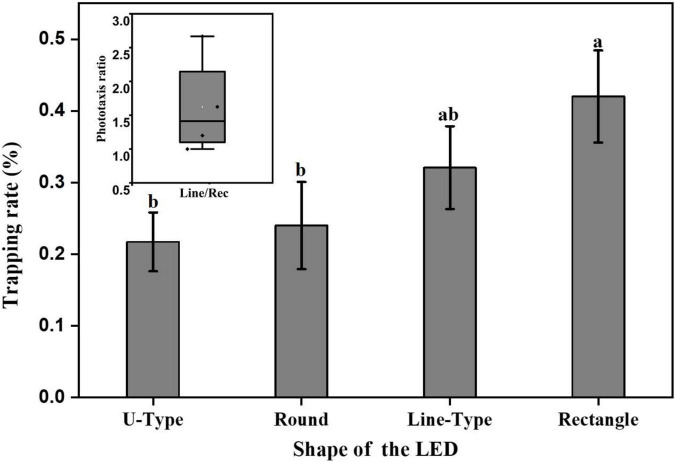
Phototaxis response rates of *L. striatellus* with different light source shapes. Each value represents the trapping rate T_1_ of the adult *L. striatellus* for that shape. Different letters above each group of data indicate significant differences between treatments (*p* < 0.01). The upper left panel shows the phototaxis rate T_2_ of linear and rectangular light sources.

**FIGURE 11:**
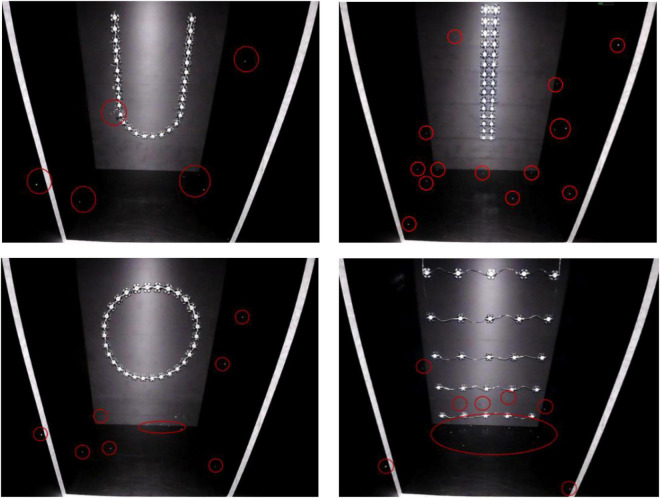
Pictures of *L. striatellus* captured by the infrared camera under four different shapes of light source (the white lines in the figure represents a distance of 20 cm from a light source). The shapes are U-shaped in the **upper left panel**, linear in the **upper right panel**, circular in the **lower left panel**, and rectangular in the **lower right panel**.

At the optimal light wavelength (460 nm), the light source shapes were optimized for *L. striatellus* phototactic behavior at a light intensity of 100 lux. The phototaxis experimental results for the three light source shapes were compared and analyzed together with the rectangular blue light source used in the light intensity experiment described in section “Analysis of the light intensity response pattern of L. striatellus phototactic behavior”. The results are shown in Figure 10. The phototropism results of rice flies under different shapes of light sources captured by the infrared camera are shown in Figure 11. All four light source shapes were able to induce a phototaxis response in *L. striatellus*. The trapping rates of *L. striatellus* in response to the rectangular and linear light sources were higher, at 41 and 31%, respectively, while the trapping rates for the U-shaped and circular light sources were lower, at only 21 and 24%, respectively. The LSD test results showed that both the rectangular and linear light sources were significantly different from the U-shaped and circular light sources (df = 3,12; *F* = 3.548; *P* = 0.047). However, the difference in phototaxis between the rectangular and linear light sources was insignificant, so the rectangular light source and the linear light source were selected for the two-channel comparison experiment. The results showed that the trapping rate of *L. striatellus* to the linear light source was on average 1.6 times that to the rectangular light source.

### Analysis of the flash frequency response pattern of the phototaxis behavior of *Laodelphax striatellus*

**FIGURE 12:**
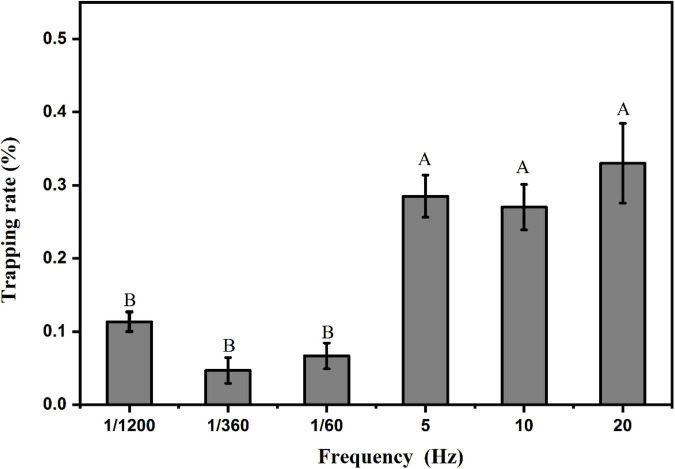
Phototaxis response rates of *L. striatellus* at different flickering frequencies. Each value represents the trapping rate T_1_ of the adult *L. striatellus* for that flash frequency of the light source. The error bars represent the standard errors. Different letters above each group of data indicate significant differences between treatments (*p* < 0.01).

Using the optimized trapping wavelength (460 nm) and trapping shape (linear shape), we optimized the flash frequency of the light source at the illumination intensity of 100 lux. The results are shown in Figure 12. We analyzed the light sources of two groups of flash frequencies, one group having three higher flash frequencies (5, 10, 20 Hz) and the other group having three lower flash frequencies (1/1200, 1/360, 1/60 Hz). The results of the F test showed that the trapping rates of *L. striatellus* were significantly higher in the high flash frequency group than in the low flash frequency group (df = 5,15; *F* = 12.721; *P* = 5.93857E-5). There were no significant differences in trapping rate among the different flash frequencies within either group.

### Analysis of combined light response patterns of *Laodelphax striatellus* phototactic behavior

**FIGURE 13:**
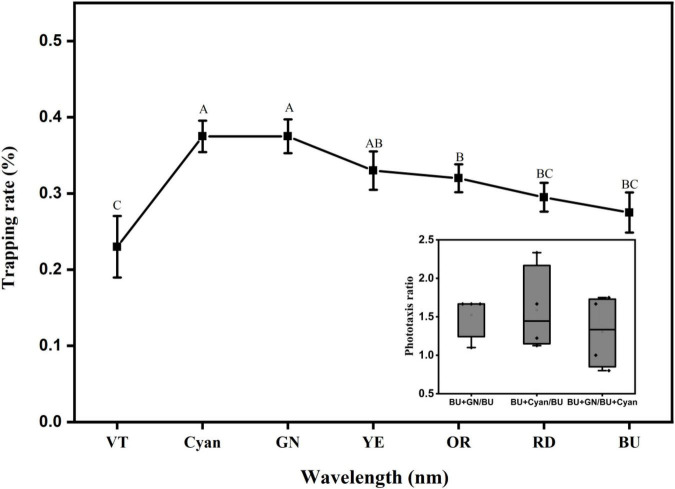
Curves of the phototaxis response rates of *L. striatellus* at different polychromatic light levels based on blue light. The horizontal coordinate represents the abbreviation of each monochromatic visible light combined with blue light (VT: violet, Cyan: cyanotype, GN: green, YE: yellow, OR: orange, RD: red, BU: blue). The BU treatment in the figure is the control blue light group. Each value represents the trapping rate T_1_ of the adult *L. striatellus* to the polychromatic light. The error bars represent the standard error. According to the LSD experiment (*p* < 0.01), different letters above each group of data indicate significant differences between treatments. The lower right panel shows the phototaxis rate T2 of blue–cyan light, blue–green light, and blue light.

With the optimized monochromatic light source, we optimized the polychromatic light combinations at a light intensity of 100 lux. The results are shown in Figure 13. The results of the F test showed that several wavelength combinations with blue light were able to induce phototactic behavior in *L. striatellus*. The trapping rates of *L. striatellus* differed significantly among different wavelength combinations (df = 6, 27; *F* = 4.259; *P* = 0.005). The trapping rate with the violet–blue combination was the lowest at 23%, which was lower than that of the blue light. The trapping rate increased with increasing wavelength. The highest trapping rates were for the blue–cyan combination and the blue–green combination, at 38%. With further increases in wavelength, the trapping rates of different combinations of light decreased. This shows that when blue light is combined with another visible light, the response to cyan and green was the best, whereas the response to violet was the worst. To further compare the phototaxis responses of the *L. striatellus* to blue–cyan and blue–green light combinations, pairwise tests for phototaxis were conducted. In the comparison tests of the two light combinations, the trapping rates of the *L. striatellus* to blue–cyan and blue–green lights were 1.5 times that to the blue light on average. In the experiment comparing the blue–cyan and blue–green lights, the trapping rate of *L. striatellus* in response to the blue–green light was 1.3 times that to the blue–cyan light on average. The response of the *L. striatellus* to polychromatic light was greater than that to monochromatic light.

## Discussion

Approaches to improve the trapping effectiveness of light sources and low-carbon light control of agricultural pests have attracted a great deal of interest. The trapping effectiveness of a light source depends on the light-induced characteristics of the target pests. A light source designed based on the light-induced characteristics of the target pests can achieve precise and efficient trapping of the target pests. The light-induced properties of pests are closely related to the photoreceptor organs—the compound eyes (Kim et al., 2019). The photoreceptor structure of the compound eyes varies greatly across different insects, and as a result, its sensitivity to wavelengths is particularly subtle in some pests (Ben-Yakir et al., 2013; Bian et al., 2018). These studies show that when optimizing the wavelength of a trapping light, attention should be paid to the precise division and screening of the wavelengths. Based on previous studies showing that *L. striatellus* are sensitive to blue light, this study conducted a more precise screening of light source wavelengths. The different T_1_ and T_2_ values of *L. striatellus* under different wavelengths indicate that the photoreceptors of a *L. striatellus*’s compound eye have bioactive behavioral excitation and selectivity in response to specific wavelengths of light stimuli. The response at 460 nm was the greatest; thus, the blue light receptors in the compound eyes of *L. striatellus* are the most developed, resulting in the greatest sensitivity to blue light (Jin, 2014). Since 460 nm is the central wavelength of the blue light band, we hypothesized that when the wavelength is closer to the central wavelength of blue light, the sensitivity increases and phototaxis becomes more active.

The light intensity of the light source determines its working range. To determine the reasonable light intensity of LED insect traps, it is important to determine the effect of the light intensity on the phototactic behavior of *L. striatellus*. Shao et al. (2013) tested phototactic behaviors of white-backed planthoppers in response to LEDs under different light intensities and found that the light intensity had no significant effect on the trapping rate of the white-backed planthopper (Shao et al., 2013). However, many studies have shown that insect phototaxis is greatly affected by light intensity (Stukenberg et al., 2015; Kim et al., 2019; Sang et al., 2019). The cause of this discrepancy may be that the light intensity gradient in study of Shao et al. (2013) was too small (10–100 lux), which makes it impossible to fully reflect the effect of light intensity on the trapping rate of *L. striatellus*. In Liu et al.’s study (2011) on the effect of the light gradient on the phototaxis response of locusts, the light intensity gradient was set from 1 to 10,000 lux, and they observed the effect of light intensity on the phototactic behavior of locusts (Liu et al., 2017). The present study expands the light intensity gradient of phototaxis experiments on the basis of existing studies(10, 20, 40, 60, 80, and 100 lux) (Shao et al., 2013) and shows that the light intensity has a significant effect on the phototaxis of *L. striatellus*. When the light intensities were low (less than 0.1 lux), the phototaxis of *L. striatellus* increased with the increase in the light intensity. When light intensity exceeded 0.1 lux, the phototaxis of the insect population no longer changed significantly. Increasing light intensity enhanced the phototaxis of *L. striatellus*, probably because that higher light intensity stimulates the photoelectric signal conversion in the retinal cells of the *L. striatellus* compound eyes and enhances their phototaxis response. However, with further increase in light intensity, a *L. striatellus* may control the amount of light entering the eyes through the movement of pigment granules on the lesser omentum cells of the compound eye, thus causing the phototaxis behavior to become stable (Ribi, 1978). It can be seen that with the increase in the light intensity gradient, the effect of the light intensity of the light source on the phototactic behavior of *L. striatellus* has been identified, and the light intensity threshold of the *L. striatellus* has been determined. In practical applications, the effect of the mounting height and placement of *L. striatellus* trapping light in the field on the actual trapping performance should also be considered to maximize the trapping effectiveness.

The shape of the light source should be considered as part of the light source design. At the same time, the shape of the target object is also an important factor in the visual perception of insects (Harris et al., 2011; Reeves, 2011). Therefore, it is also important to study the effect of the shape of the light source on *L. striatellus*. This study conducted the first experiments on *L. striatellus* with different light source shapes. The results of the single-channel and dual-channel experiments showed that the linear light source performed better than the circular, U-shaped, and rectangular light sources. The different T_1_ and T_2_ values for different light source shapes probably indicated that the photoreceptors in the compound eyes of *L. striatellus* had different neural excitations to light stimulation induced by light source shapes. Studies have found that this may be related to the ability of insects to discriminate the shape of target objects in the selection of their host plants during evolution (Byrne et al., 1986). We hypothesized that since *L. striatellus* live on elongated rice stems for a long time, they have the best phototaxis response to linear light sources. Bian et al. (2018) designed light sources with three shapes: cylindrical, hemispherical, and tubular, conducted trapping experiments on several major pests at a tea farm, and showed that the cylindrical light source performed the best (Bian et al., 2018). In explaining the mechanism of this phenomenon, this paper innovatively links it to the shape of the host plant selected by insects. Some studies have found that the shape and structure of host plants can sometimes strongly stimulate the vision of insects and can produce considerable trapping effects on some insect species (Liu and Zhao, 2017). *L. striatellus* live in the stems of rice for long periods of time. The stems are long and thin, so the *L. striatellus* have better tropism to long and thin cylindrical light sources. In addition, studies have found that cylindrical light sources have relatively good trapping effectiveness on *Liriomyza sativae* and *Bemisia tabaci* (Byrne et al., 1986; Jiang et al., 2007). It can be seen that elongated light sources are widely preferred in different insect populations, which is consistent with the results of this study. Our findings provide a theoretical basis for promoting the light trapping of other pests using linear light sources.

In our study we also tested the attractiveness of light sources for trapping insects under different flash frequencies. The results showed that the trapping rates in the higher flash frequency group were significantly higher than those in the lower flash frequency group but was approximately the same as that of the non-flashing light source. The reason for this result is probably that the illumination time of the lower flash frequency group was too short to form a continuous illumination stimulus. As a result, a complete photoelectric signal circuit could not be formed in the retinal cells of the compound eye, so it could not be stimulated to produce phototaxis. In the higher flash frequency group, the flash frequencies caused continuous stimulation to the compound eyes of the *L. striatellus* and stimulated phototaxis, so the trapping rates were generally higher. It is worth noting that the trapping rates of the high flash frequency light sources were not significantly higher than those in response to the non-flashing light sources. Although it will reduce power consumption, it will increase the complexity and cost of the circuit, it also will reduce the service life of the light source. So it is not recommended to use pest control with flashing lights during rice growth.

The development of LED-light sources for insect trapping based on multiple light color combinations is rarely reported, likely because of the complexity of insect responses to polychromatic light. In this study, we combined different visible color lights with blue lights. Through single-channel and dual-channel experiments, we conducted a phototaxis response experiment of *L. striatellus* to polychromatic light and optimized the wavelength combination of the light source. The results showed that the trapping rates for polychromatic lights were significantly improved compared with that of monochromatic light, and the trapping rate for the combined blue-green color light was 1.5 times that of the blue light (Figure 13). We hypothesized that with different visible light and blue light combinations, the neural excitation of photoreceptors in the compound eyes of *L. striatellus* may differ, resulting in different phototactic behaviors of the *L. striatellus*. Multiple neural response channels might have a superimposed effect on the neural responses generated by polychromatic light stimulation (Jander and Barry, 1968), thereby increasing the phototaxis response of the insects. This finding provides a theoretical basis for the application of combined light sources in *L. striatellus* traps. Ding (1978) observed that when trapping *Heliothis assulta* adults, the combinations of 350 with 405 nm and 350 with 436 nm lights enhanced the performance, while the combination of 350 nm with 578–656 nm lights had an interference and repelling effect (Ding, 1978). This indicates that for a specific light source, an insect not only exhibits positive phototactic behavior but also negative phototactic behavior. In this study, the trapping rate of combined blue–violet light was significantly lower than that of blue light, indicating that purple may have a repelling effect on *L. striatellus* (Figure 13). In a phototaxis study of pests and natural enemies in tea farms, it was found that the natural enemies of many pests, including flat-bellied wasps and trichogramma, often move to dark areas under certain wavelengths to avoid light (Bian et al., 2018). Therefore, adding an effective light band that repels natural enemy species of *L. striatellus* in the combined light will likely reduce the harm caused by the light source to the natural enemy population, thereby improving the safety and trapping effectiveness of the light source. This may be a direction in the research of polychromatic light sources for insect trapping.

It is noteworthy that under the present experimental conditions, the phototropism of the *L. striatellus* population was always below 50%, regardless of the variation of light source parameters. We speculate that this may be related to the physiological function of the insect population. It was found that the sex, age, mating status, and whether the insects were hungry or not would affect their phototropism behavior (Kim et al., 2019). Therefore, a large number of physiological experiments are needed to better unlocking the secrets of insect phototropism.

## Conclusion

In this study, we conducted phototactic experiments on *L. striatellus* following specific steps and using single-channel and double-channel experiments to determine the response patterns of *L. striatellus* to the wavelength, intensity, shape, and flash frequency of monochromatic light, as well as color combination of light sources. The results of the experiments showed that these factors can significantly affect the phototaxis of *L. striatellus*, thus affecting the trapping rate of the population. The sensitivity of *L. striatellus* to the wavelength of the monochromatic light source was significantly different between 440 and 500 nm, and the phototaxis increased first and then decreased with increasing wavelength. The sensitivity peaked at 460 nm, at which the phototaxis values were 1.3 times that at 450 nm and 1.5 times that at 470 nm. The phototactic behavior of *L. striatellus* was affected by the light intensity. When the light intensity was less than 0.1 lux, the trapping rate increased with the light intensity and leveled off when the light intensity exceeded 0.1 lux. In addition, the light sources of different shapes also had a significant effect on the phototaxis of *L. striatellus*. The results showed that the phototaxis of the linear light source was the highest at 460 nm. Based on the above results on *L. striatellus* phototaxis to monochromatic light sources, this study also explored the response characteristics of *L. striatellus* to combined light and compared them with 460 nm linear-type light sources. Our experiments showed that compared with monochromatic light, the phototaxis of *L. striatellus* was significantly improved by polychromatic light, and the improvement with blue–green combined light was the best, reaching 1.5 times that of monochromatic light. In this study, through the step-by-step phototaxis experiment, we determined that the best light source for trapping *L. striatellus* is a blue–green light source, and we identified metrics in terms of the light intensity, shape, and flash frequency of the light source. This study provides a theoretical basis and data support for the development of LED light source for trapping *L. striatellus*.

## Data availability statement

The original contributions presented in the study are included in the article/supplementary material, further inquiries can be directed to the corresponding author.

## Author contributions

SZ: data curation. SZ and KX: formal analysis. JN: funding acquisition. SZ and JW: investigation. JN and SZ: methodology and writing—review and editing. ZS and YL: hardware. JN: project administration. WC and YT: resources and supervision. JN, KX, and SZ: validation. SZ: writing—original draft. All authors have read and agreed to the published version of the manuscript.
